# Acceleration of bone regeneration of horizontal bone defect in rats using collagen‐binding basic fibroblast growth factor combined with collagen scaffolds

**DOI:** 10.1002/JPER.18-0674

**Published:** 2019-04-14

**Authors:** Shin Nakamura, Takashi Ito, Kentaro Okamoto, Takehiko Mima, Kentaro Uchida, Yasir D. Siddiqui, Masahiro Ito, Masako Tai, Keisuke Okubo, Keisuke Yamashiro, Kazuhiro Omori, Tadashi Yamamoto, Osamu Matsushita, Shogo Takashiba

**Affiliations:** ^1^ Department of Pathophysiology‐Periodontal Science Dentistry and Pharmaceutical Sciences Okayama University Graduate School of Medicine Okayama Japan; ^2^ Department of Bacteriology Dentistry and Pharmaceutical Sciences Okayama University Graduate School of Medicine Okayama Japan; ^3^ Ministry of Health Labour and Welfare Medical Politics Economic Section Medical Equipment Policy Office Tokyo Japan; ^4^ Department of Orthopedic Surgery Kitasato University School of Medicine Sagamihara, Kanagawa Japan; ^5^ Department of Periodontics and Endodontics Okayama University Hospital Okayama Japan

**Keywords:** bone regeneration, collagen, drug delivery systems, growth factors, periodontitis, tissue engineering

## Abstract

**Background:**

Basic fibroblast growth factor (bFGF) has been applied for periodontal regeneration. However, the application depends on bone defect morphology because bFGF diffuses rapidly from defect sites. In a previous study, collagen‐binding bFGF (CB‐bFGF) has been shown to enhance bone formation by collagen‐anchoring in the orthopedic field. The aim of this study is to demonstrate the efficacy of CB‐bFGF with collagen scaffolds in bone regeneration of horizontal bone defect.

**Methods:**

Cell proliferation activity and collagen binding activity of CB‐bFGF was confirmed by WST‐8 assay and collagen binding assay, respectively. The retention of CB‐bFGF in the collagen sheet (CS) was measured by fluorescence imaging. The rat horizontal alveolar bone defect model was employed to investigate the efficacy of CB‐bFGF with collagen powder (CP). After 4 and 8 weeks, the regenerative efficacy was evaluated by microcomputed tomography, histological, and immunohistochemical analyses.

**Results:**

CB‐bFGF had a comparable proliferation activity to bFGF and a collagen binding activity. CB‐bFGF was retained in CS longer than bFGF. At 8 weeks postoperation, bone volume, bone mineral content, and new bone area in CB‐bFGF/CP group were significantly increased compared with those in other groups. Furthermore, epithelial downgrowth was significantly suppressed in CB‐bFGF/CP group. At 4 weeks, the numbers of osteocalcin, proliferating cell nuclear antigen, and osteopontin‐positive cells at the regeneration site in CB‐bFGF/CP group were greater than those in other groups.

**Conclusions:**

CB‐bFGF/CP effectively promoted bone regeneration of horizontal bone defect possibly by sustained release of bFGF. The potential of CB‐bFGF composite material for improved periodontal regeneration in vertical axis was shown.

## 1 INTRODUCTION

Periodontitis is an inflammatory disease that causes destruction of periodontal tissues as a result of a complex interplay between bacterial infection and host immune response.^1^ The most common treatment of periodontitis is mechanical removal of bacterial biofilm by scaling and root planing.^2^ To treat severe periodontitis, a surgical approach aims to remove the source of infection by flap operation.^3^ However, due to invasion of the epithelial tissue into the alveolar bone defect site, it is difficult to regenerate periodontal tissues such as alveolar bone and periodontal ligament, and the risk of recurrence of periodontitis remains. In ideal periodontal tissue regeneration, minimum epithelial adhesion, the formation of new cementum enclosing collagen fibers, and formation of new bone are required.^4^ Therefore, periodontal regeneration therapy that promotes restoration of the structure and function of the destroyed periodontal tissue has been used, such as guided tissue regeneration (GTR) treatment^5^ and the application of an enamel matrix derivative (EMD),^6^ and the application of various growth factors.^7^, ^8^, ^9^

Moreover, basic fibroblast growth factor (bFGF) is a growth factor that is known to promote fibroblast cell proliferation and bone formation.^10^, ^11^ bFGF promotes the formation of new periodontal tissue when applied in a dog alveolar bone defect model,^12^, ^13^ and significantly enhances new alveolar bone formation in chronic periodontitis patients in randomized clinical trials.^14^, ^15^

However, bFGF has low target specificity and a short retention time in the local tissue.^16^, ^17^ For sustained therapeutic effect, large doses of bFGF are required, which raises the risk of adverse side effects.^18^ Furthermore, in periodontal tissue regeneration therapy, the administered drugs tend to be lost because teeth are implanted through the epithelium, and the surgical site easily becomes a wound window after surgery. Indeed, especially in horizontal bone loss or severe furcation involvement, it is difficult for conventional therapeutics to maintain a high concentration of growth factors sufficient for local tissues to induce periodontal tissue regeneration. To overcome these anatomical characteristics of the oral environment, it is important for therapeutics to provide higher efficacy by being retained in the local tissue.^19^

Therefore, we focused on the collagen‐anchoring of growth factors. Myonecrosis is a life‐threatening infection caused by histotoxic clostridia. They commonly produce collagenases to hydrolyze insoluble collagen fibers to rapidly expand the infection foci. *Clostridium histolyticum* is one of the causative microbes, which produces two classes of collagenases.^20^, ^21^ They possess collagen anchors at their C‐termini to anchor themselves on collagen fibers for efficient hydrolysis.^22^, ^23^, ^24^, ^25^, ^26^ Nishi et al.^27^ constructed bFGF fused with one of the collagen anchors (CB‐bFGF) to show that CB‐bFGF, but not bFGF, strongly stimulated the DNA synthesis in stromal cells at 5 and 7 days after injection when injected subcutaneously into mice. Moreover, the efficacy of CB‐bFGF for osteogenesis has been reported in the orthopedic field. Using a mouse femur fracture model, it was reported that CB‐bFGF significantly promotes bone formation compared with bFGF when anchored in various collagen scaffolds including collagen sheets (CS),^28^, ^29^ collagen powder (CP),^30^ collagenous peptide gel,^31^ and demineralized bone.^32^ Clostridial collagenase has already been commercialized, and its biological safety is guaranteed.^33^, ^34^ Hence, the collagen anchor, which is a part of collagenase, is also expected to be safe for clinical application.

We assumed that this prolonged bFGF efficacy by collagen‐anchoring is applicable to establishing a novel periodontal regeneration therapy. Whereas, in the case of applying these composite materials to periodontal therapy, it is necessary to overcome the anatomical barriers specific for periodontal surgery described above. Thus, in this study, we evaluated the efficacy of composite material for bone regeneration at the periodontal tissue.

## 2 MATERIALS AND METHODS

### 2.1 Animals

Male Sprague‐Dawley rats^1^ (10 to 12‐weeks‐old) were used. This study was approved by the Animal Care and Use Committee at Okayama University (Permit no: OKU‐2017054). All animal experiments were performed in accordance with the Guidelines for Animal Experiments of Okayama University, surgical procedures were performed under xylazine (10 mg/kg) and ketamine (100 mg/kg), and all efforts were made to minimize animal suffering.

### 2.2 Test substances

Human bFGF was purchased from Miltenyi Biotec (Tokyo, Japan). Fusion protein (CB‐bFGF) composed of bFGF and collagen‐binding‐domain derived from *C. histolyticum* class II collagenase (ColH) was produced using the method described previously.^27^ Briefly, GST‐tagged CB‐bFGF was produced in *Escherichia coli* BL21 strain^2^ harboring the plasmid (pCHC302‐hbFGF). GST‐tagged CB‐bFGF was purified by affinity chromatography with glutathione sepharose.^3^ After the GST tag was cleaved by thrombin,^4^ CB‐bFGF was purified by affinity chromatography with heparin sepharose.^5^ CP and CS were provided by Nippi (Ibaraki, Japan).

### 2.3 Cell proliferation assay

Cell proliferation assay was performed using a method described previously.^35^, ^36^ The lower incisors of the rats were extracted, and the periodontal ligament tissue attached to the root surface was collected. The collected periodontal ligament tissue was treated with 2 mg/mL collagenase type I^6^ and 4 mg/mL dispase^7^ for 60 minutes. The enzyme‐treated tissue was washed with phosphate buffered saline (PBS), pH 7.2.^8^ We cultured cells in minimum essential medium eagle, alpha modification (αMEM)^9^ containing 10% fetal bovine serum (FBS)^8^ at 37°C, 5% CO_2_, and 95% humidity. After 3 to 5 passages, cells were seeded in αMEM containing 2% FBS on a 96‐well plate at a density of 1.0 × 10^3^ cells/ well. After 5 hours of incubation, bFGF or CB‐bFGF were added at concentrations of 1, 10, 100, and 1,000 pM. The WST‐8 assay was performed using cell counting kit^10^ at 72 hours after bFGF addition to assess the cell proliferation.

### 2.4 Collagen binding assay

A collagen binding assay was performed using a method described previously.^22^ Briefly, 0.4 nmol of bFGF or CB‐bFGF was mixed with 5 mg of CP and incubated at 4°C for 30 minutes. After centrifugation at 12,000 rpm for 4 minutes, the supernatant containing proteins that did not bind to CP was analyzed by sodium dodecyl sulfate polyacrylamide electrophoresis (SDS‐PAGE).

### 2.5 Comparison of retention of fluorescently labeled proteins in the collagen sheet

bFGF or CB‐bFGF (6 µM) was prepared in 50 mM Tris‐HCl, pH 7.5 containing 1 mM CaCl_2_. Then, 10 µL of 1 mM Tris(2‐carboxyethyl)phosphine^11^ was added and incubated at 37°C for 30 minutes. Subsequently, 10 µl of 1 mM fluorescence dye^12^ was added and incubated overnight at 4°C. The sample was loaded on a spin column^13^ and centrifuged at 800×g at 4°C for 2 minutes to remove unreacted dye. Fluorescence labeling was confirmed by SDS‐PAGE, followed by photographing with a software.^14^ Labeled bFGF or CB‐bFGF (0.58 nmol) was mixed with 5 × 5 × 2 mm (length × width × depth) of CS for 30 minutes and centrifuged at 12,000 rpm for 4 minutes. Binding to the CS was confirmed by analyzing the supernatant by SDS‐PAGE. The bFGF‐ or CB‐bFGF‐bound CS was placed in a 12‐well plate containing 1‐mL αMEM. After 0, 1, 3, 5, 10, and 14 days, fluorescence imaging was performed by the IVIS system.^15^ The area of the fluorescence detection range on the CS was analyzed using a public domain software.^16^

### 2.6 Bone defect model

**Figure 1:**
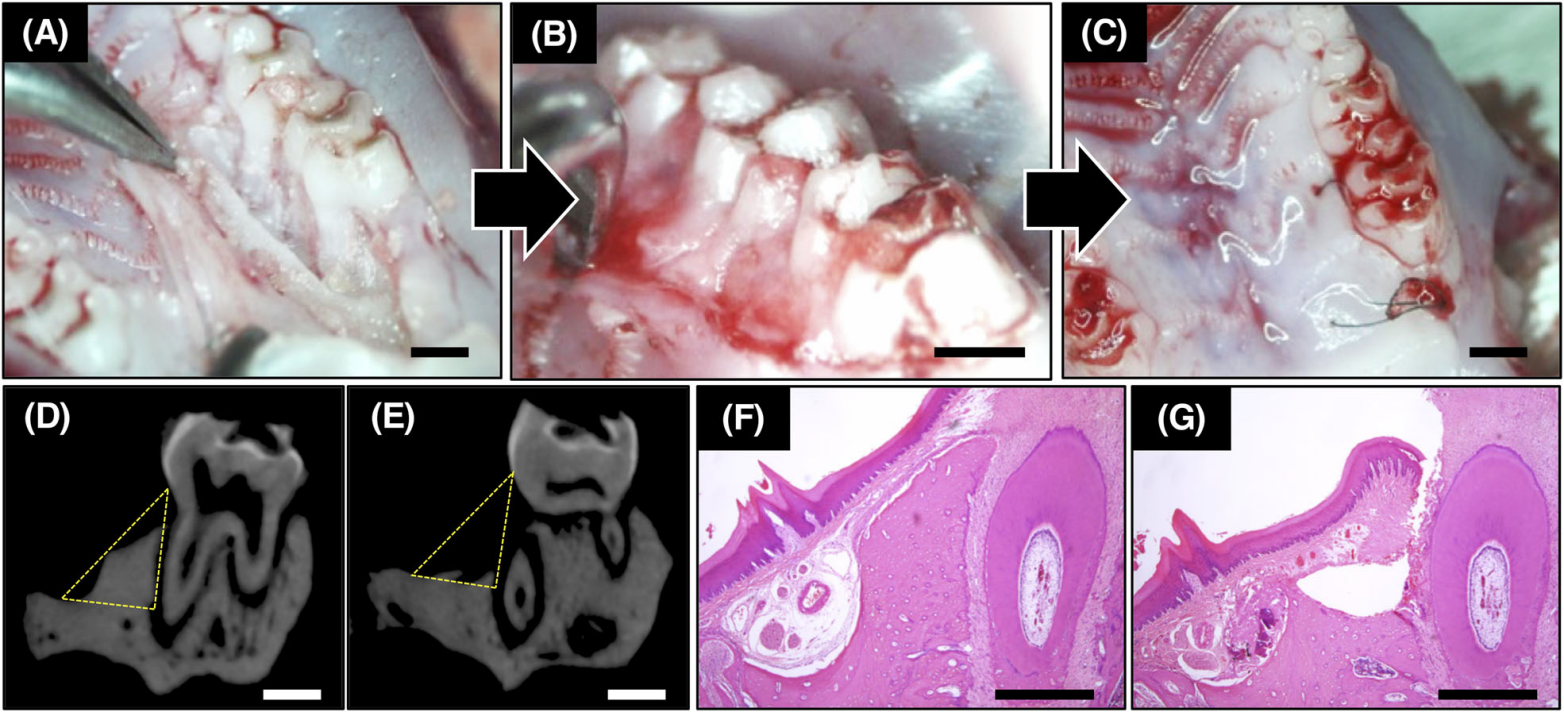
Creation of bone defect model. **A**) Bone defect created on the first molar palatal side after raising the full‐thickness flap. **B**) Loading test substance. **C**) Closed operation site by suture. Scale bars in Panel A to C indicate 1.0 mm. **D** and **E**) Micro‐CT images of untreated side and bone defect side at day 0. Bone volume and bone mineral content within the yellow range were analyzed. Scale bars in Panels D and E indicate 1.0 mm. **F** and **G**) H&E staining of untreated side and bone defect side at day 0. Scale bars in Panels F and G indicate 500 µm

The rat maxillary palatal gingiva was incised and peeled from the mesial first molar to the distal second molar. A horizontal bone defect was created by drilling 1.0 mm on the palatal alveolar bone using a tungsten carbide bar^17^ (Fig. 1A). CP (5 mg) mixed with 0.58 nmol of bFGF or CB‐bFGF was applied to the defect site (Fig. 1B). The loading volume was based on previous studies.^30^ Finally, the operation site was sutured with 8.0 nylon thread (Fig. 1C). After 4 and 8 weeks, the rats were euthanized by CO_2_, and the bone formation was evaluated by microcomputed tomography (micro‐CT) and histology.

### 2.7 Quantification of bone volume and bone mineral content

Rat maxillary bone was excised and fixed in 4% phosphate buffered paraformaldehyde^18^ and stored at 4°C for 72 hours. Micro‐CT images were obtained using a microfocus x‐ray CT imaging system.^19^ Images were obtained using the following settings: acceleration voltage, 90 kV; current, 110 mA; voxel size, 20 µm/pixel; matrix size, 1,024 × 1,024. Images of the bone defect site were obtained and 2.4 mm regions of interest (120 slices) were defined from the contact point between the first and second molar to the mesial first molar. Bone volume and bone mineral content were measured in the region surrounded by the base line of the palatine bones and a perpendicular line drawn along the root toward the crown including existing bone and new bone (Figs. 1D and 1E, yellow area) by using three‐dimensional (3D) image analysis software.^20^

### 2.8 Histological evaluation

After fixation, the samples were decalcified with 10% formic acid for 10 days and then embedded in paraffin. Then, 4‐µm sections were sliced from the first molar mesial cusp toward the distal direction and evaluated by hematoxylin‐eosin staining (H&E staining) and Azan staining. In the H&E stained section, the new bone area was quantified by a public domain software^21^ (Figs. 1F and 1G). New bone formation was defined as the area identified trabecula and bone marrow based on the surgical margins of the cortical bone. For each sample, three slices were prepared every 50 µm, and the average value was calculated. In Azan staining, the length of epithelial adhesion was measured using a public domain software.^21^

### 2.9 Immunohistochemistry and quantitative analysis

For immunohistochemistry, we used an avidin‐biotin‐peroxidase complex method.^22^ Sections were deparaffinized with xylol, immersed in 0.3% H_2_O_2_/methanol, and antigen was activated by trypsin.^23^ Subsequently, they were blocked with normal serum solution and reacted with the primary antibodies overnight at 4°C. Rabbit polyclonal antibody against osteocalcin (OCN)^24^ at a dilution of 1:500 in PBS, rabbit polyclonal antibody against proliferating cell nuclear antigen (PCNA)^25^ at a dilution of 1:100 in PBS, and rabbit polyclonal antibody against osteopontin (OPN)^24^ at a dilution of 1:100 in PBS were used. As negative controls, some sections were incubated without primary antibody. The sections were incubated for 30 minutes at 25°C with the secondary antibodies, goat anti‐rabbit antibodies, at a dilution of 1:200. They were treated with peroxidase‐linked streptavidin solution for 30 minutes at room temperature and were treated with diaminobenzidine (DAB)^27^ solution containing 0.02% H_2_O_2_. After counterstaining with hematoxylin, DAB signals were observed under light microscopy. The number of positive cells was measured around the new bone and root surface at the regeneration site. We chose at random from three sections from each rat and counted positive cells.

### 2.10 Statistical analysis

All of the data are presented as the means ± SD. One‐way ANOVA with Tukey‐Kramer multiple comparison test was used to examine differences among the control, PBS/CP, bFGF/CP, and CB‐bFGF/CP groups. For the analysis between the two groups, a student *t*‐test was conducted. For each statistical process, a test was carried out using software,^26^ and a *P* value of <0.05 was considered statistically significant.

## 3 RESULTS

### 3.1 Characterization of CB‐bFGF in vitro

**Figure 2:**
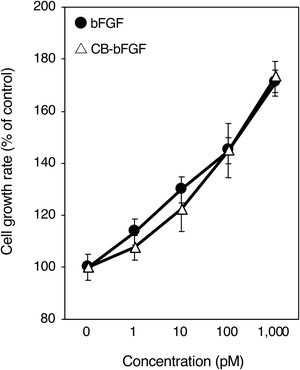
Dose‐response curves for the growth factor activity of bFGF and CB‐bFGF. Periodontal ligament cells were inoculated onto 96‐well plates, and 5 hours later test substances were added. After 72 hours, a WST‐8 assay was performed, and the cell growth rate was calculated (percentage of control). Each point represents the mean value ± SD for triplicate experiments

**Figure 3:**
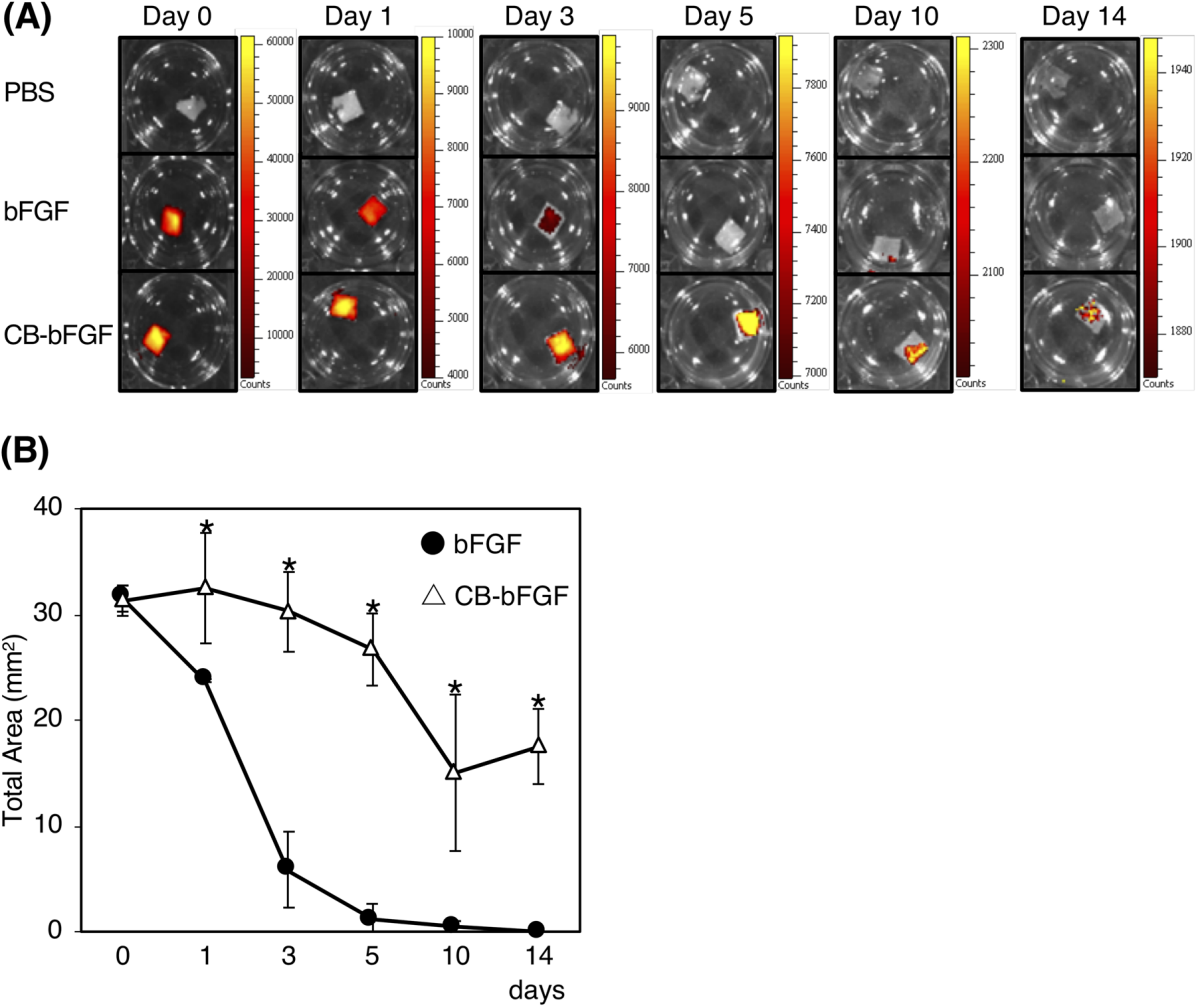
Comparison of retention of fluorescently labeled proteins in collagen sheet. **A**) Typical images of the signal intensity of the bFGF or CB‐bFGF in CS. **B**) Total area of the signal intensities on CS. Student *t‐*test was used to compare both. Data are presented as the mean ± SD (n = 3). *P < 0.05

The in vitro properties of purified CB‐bFGF were examined. According to the cell proliferation assay, purified CB‐bFGF showed the same degree of cell proliferation activity as bFGF in the range of 1 to 1,000 pM (Fig. 2). When the supernatant of CB‐bFGF or bFGF solution after mixing with CP was analyzed by SDS‐PAGE, the band of CB‐bFGF was not observed, whereas that of bFGF was observed (see Supplementary Figure 1 in online *Journal of Periodontology*). This result shows the collagen binding activity of CB‐bFGF. The collagen binding activity of CB‐bFGF was further confirmed using fluorescent‐labeled protein (see Supplementary Figure 2 in online *Journal of Periodontology*). The retention of CB‐bFGF in CS was evaluated using the fluorescent‐labeled protein. Although the fluorescence intensity of both bFGF and CB‐bFGF decreased gradually, the area of the fluorescence detection of CB‐bFGF was significantly larger than that of bFGF for 14 days (Figs. 3A and 3B).

### 3.2 In vivo bone volume and bone mineral content analysis

**Figure 4:**
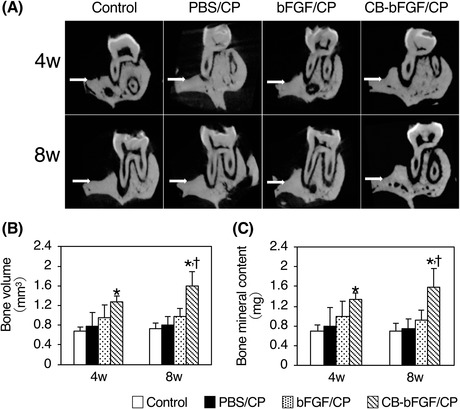
Micro‐CT analysis of bone defect site at 4 and 8 weeks after operation. **A**) Two‐dimensional images of representative samples. White arrows show the baseline of the horizontal bone defect site. Quantitative analysis of bone volume (**B**) and bone mineral content (**C**) at the bone defect site. Analysis range is shown in Figures 1D and 1E. Data shown as mean ± SD (n = 5) by one‐way ANOVA and Tukey‐Kramer test. *P < 0.05 compared with control group. ^†^ *P* < 0.05 compared with bFGF/CP group

In the group loaded with CB‐bFGF/CP, bone formation at the defect site was observed compared with the other groups (Fig. 4A). At 4 weeks after loading CB‐bFGF/CP, both bone volume and bone mineral content were significantly increased compared with the control group. In comparison with the bFGF/CP group, there was a tendency to increase both bone volume and bone mineral content. In contrast, after 8 weeks, the CB‐bFGF/CP group exhibited significantly increased bone volume and bone mineral content compared with the other groups, and those in the CB‐bFGF/CP group were about 1.5 times those of the bFGF/CP group (Figs. 4B and 4C).

### 3.3 Histomorphological evaluation

**Figure 5:**
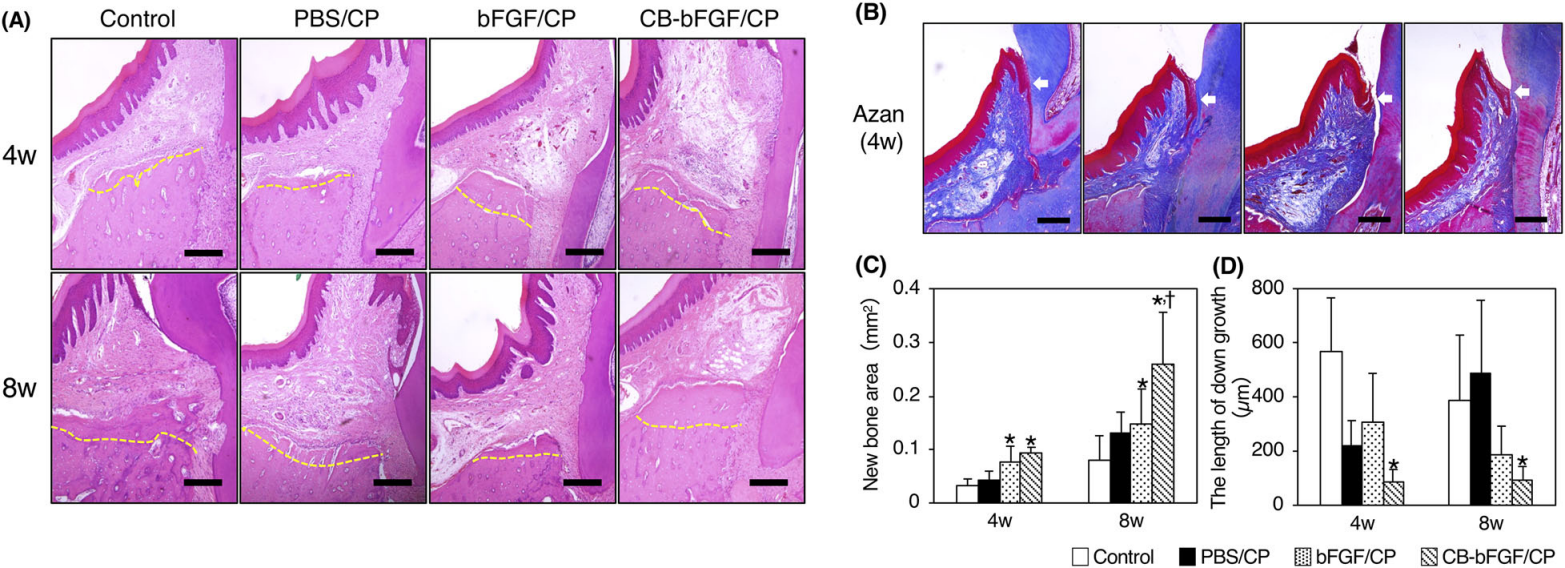
Quantification of new bone area. **A**) Histological overview at 4 and 8 weeks postoperation. Yellow dotted line represents the original bone defect level. Scale bars indicate 100 µm. **B**) Histological images of the downgrowth of the epithelial tissues at 4 weeks (white arrows). Scale bars indicate 100 µm. **C**) The data indicating new bone area are presented as mean ± SD (n = 5) by one‐way ANOVA and Tukey‐Kramer test. The measurement of the length of downgrowth of epithelial tissue. **D**) The data indicating the length of the downgrowth of epithelial tissue are presented as the mean ± SD (n = 5) by one‐way ANOVA and Tukey‐Kramer test. *P < 0.05 compared with control group. ^†^ *P* < 0.05 compared with bFGF/CP group

The operative site was evaluated by H&E staining. The new bone formation was confirmed at 4 and 8 weeks (Fig. 5A). Quantification of new bone area in H&E‐stained sections showed that in the bFGF/CP group and CB‐bFGF/CP group, the new bone area was significantly increased after 4 weeks compared with the control group. At 8 weeks postoperation, the new bone area in the CB‐bFGF/CP group was significantly increased compared with the other groups, and it was about 1.5 times that of the bFGF/CP group (Fig. 5C).

The operative site was also evaluated by Azan staining (Fig. 5B). Although the downgrowth of epithelial tissues was observed in all samples in the control group, those samples were decreased by loading test substances. Measurement of the length of epithelial adhesion showed that, in the CB‐bFGF/CP group, the length of downgrowth was significantly shorter after 4 and 8 weeks compared with the control group (Fig. 5D).

### 3.4 Localization and quantification of OCN‐, PCNA‐, and OPN‐positive cells at the operative site

**Figure 6:**
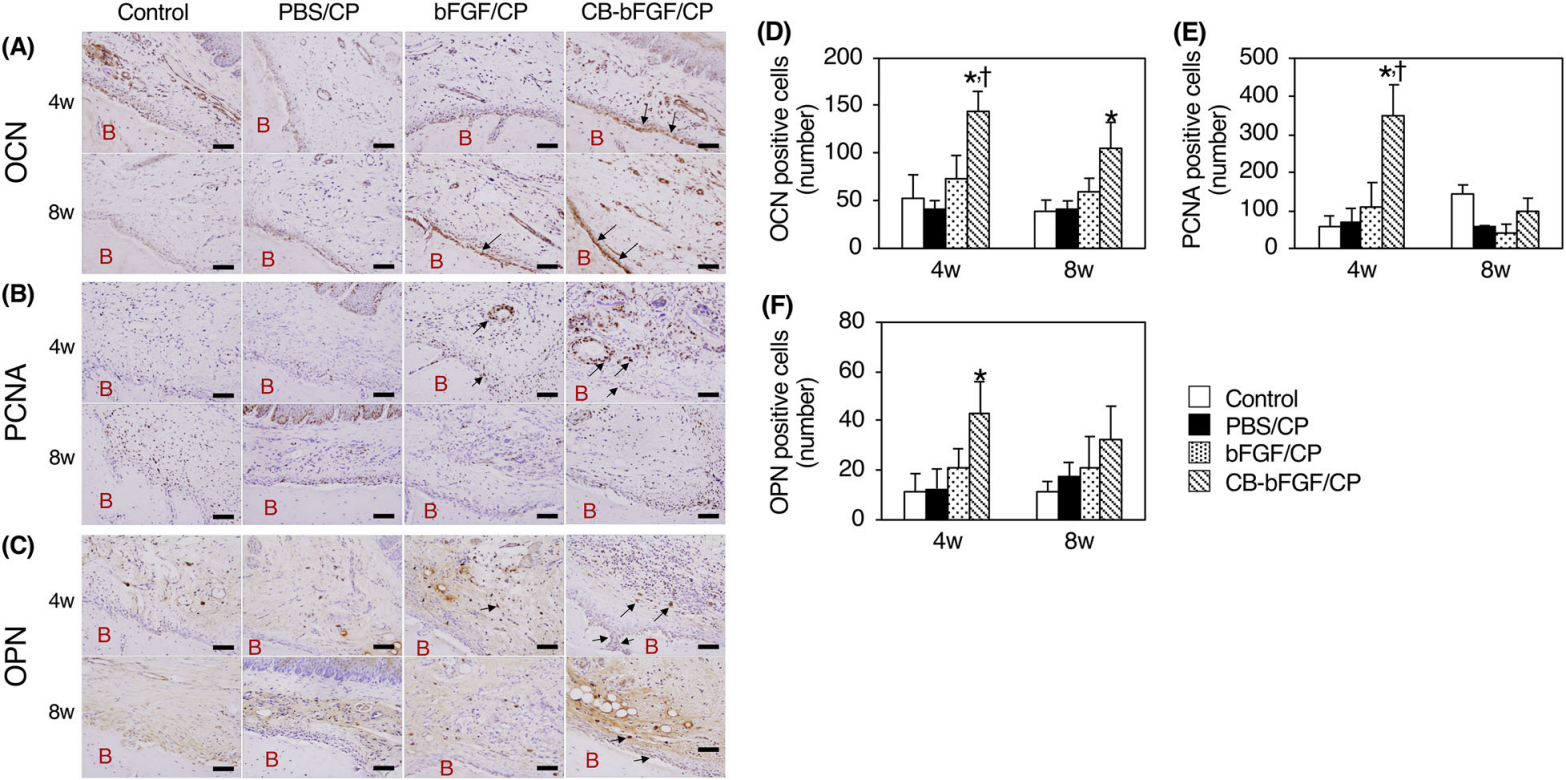
Localization of OCN, PCNA, and OPN positive cells and quantitative analysis. Representative images of the immunohistochemical staining at 4 and 8 weeks (**A** through **C**). Scale bars indicate 100 µm. B: New bone. Arrows: Positive cells. Quantification of the number of the positive cells (**D** through **F**). Data shown as mean ± SD (n = 3) by one‐way ANOVA and Tukey‐Kramer test. *P < 0.05 compared with control group. ^†^ *P* < 0.05 compared with bFGF/CP group

OCN‐positive cells were prominently observed on the surface of new bone in the CB‐bFGF/CP group (Fig. 6A). The number of positive cells was significantly increased compared with the other groups at 4 weeks. Although the number of OCN‐positive cells was reduced at 8 weeks, it was significantly higher than that of the control group (Fig. 6D). PCNA‐positive cells were clearly observed in the connective tissue adjacent to the root surface and newly formed bone side in the CB‐bFGF group at 4 weeks (Fig. 6B). The number of positive cells was significantly increased compared with the other groups. After 8 weeks, PCNA‐positive cells were reduced and there was no significant difference in either group (Fig. 6E). OPN‐positive cells were prominently observed in connective tissue adjacent to the new bone surface in the CB‐bFGF/CP group at 4 weeks (Fig. 6C). The number of positive cells was significantly increased compared with the control group. However, after 8 weeks, OPN‐positive cells were reduced and there was no significant difference in either group (Fig. 6F).

## 4 DISCUSSION

bFGF has been applied clinically as a periodontal tissue regeneration therapeutic drug. However, its application is limited to vertical bone defect, and it is difficult to apply for complex bone defects including horizontal bone defect. Therefore, we evaluated the efficacy of a composite material made of bFGF fused with a collagen anchor and collagenous scaffolds (CB‐bFGF/CP) for bone regeneration at the periodontal tissue.

Before the composite material was evaluated in vivo, properties of purified CB‐bFGF were confirmed by in vitro assays. Cell proliferation activity comparable with bFGF was confirmed using periodontal ligament cells. It has been reported that periodontal ligament cells are key players during periodontal tissue regeneration.^37^ This result suggests that CB‐bFGF is no less suitable than bFGF for periodontal regeneration. Collagen binding activity was confirmed by collagen binding assay with CP. We selected CP as an appropriate collagen scaffold to transplant periodontal tissues because it is easy to operate and adapt to the surgical site. We also examined the retention of bound bFGF quantitatively in vitro by fluorescent imaging. An alternative scaffold (CS) was used for a more accurate measurement in our imaging system. The result clearly showed a prolonged binding of the bFGF moiety in the scaffold in this model. Since CP and CS were made of porcine type I collagen, retention of CB‐bFGF in both materials is presumed to be comparable.

After the properties of CB‐bFGF were confirmed by in vitro assays, the rat alveolar bone defect model was used to examine the efficacy of CB‐bFGF/CP in vivo. This model simulated general periodontal surgery for horizontal bone defects. The result of micro‐CT and histomorphometric analyses showed that bone formation after 8 weeks was significantly promoted in the CB‐bFGF/CP group compared with the other groups. This results suggest that CB‐bFGF was retained at the local tissue by binding to the scaffold to provide higher efficacy. These findings have important implications for the clinical application of CB‐bFGF/CP for bone regeneration.

It is important to promote the formation of new bone, cementum, and periodontal ligament while suppressing the invasion of epithelial tissue. During the initial stages of periodontal tissue regeneration, bFGF promotes the proliferation of fibroblastic cells, and subsequently, the fibroblastic cells differentiate into osteoblasts, cementoblasts, and periodontal ligament cells, inducing marked periodontal regeneration.^38^ In our study, downgrowth of epithelial tissue was significantly suppressed by loading CB‐bFGF/CP at 4 and 8 weeks compared with the control groups. CB‐bFGF/CP may lead to the formation of new periodontal tissues more than bFGF by its sustained release from the collagen scaffold in vivo.

Moreover, immunohistochemistry was performed to examine the localization and time frame of cells responsible for the formation of new periodontal tissues at the defect site 4 and 8 weeks after surgery. OCN is a calcium binding protein secreted solely by osteoblasts and plays an important role in the process of ossification for bone formation in periodontal tissue.^39^ It is commonly used as a marker of osteogenic differentiation from osteoblasts.^40^ In the CB‐bFGF/CP group, the number of positive cells was greater than that in other groups at 4 weeks, and was greater than the control group after 8 weeks at the new bone surface. This result suggests that CB‐bFGF/CP promotes bone formation as early as at 4 weeks and sustains it for 8 weeks. PCNA acts as a cofactor for DNA polymerases involved in DNA replication, and hence it is a useful marker for the DNA synthesis phase of the cell cycle.^41^ PCNA‐positive cells were observed in connective tissue and periodontal ligament tissue, and the number of cells in the CB‐bFGF/CP group at 4 weeks was increased compared with other groups. These cells were fibroblastic cells derived from bone marrow and periodontal ligament. It is thought that CB‐bFGF/CP promoted the proliferation of these cells to contribute to the formation of new periodontal tissues. OPN is an extracellular acidic protein that binds to hydroxyapatite with Arg‐Gly‐Asp motifs, which binds to integrin. It is essential for normal early callus formation and neovascularization.^42^ In our model, OPN‐positive cells were observed at the new bone surface and connective tissue, and the number of OPN‐positive cells in CB‐bFGF/CP at 4 weeks was greater than that in the control group. The positive cells on the bone surface were likely osteoblasts or osteoclasts, and the cells in connective tissue are likely cells involved in angiogenesis and extracellular matrix (ECM) mineralization. In summary, these results indicate that CB‐bFGF/CP promotes bone remodeling and wound healing as early as 4 weeks postoperation, and the formation of new periodontal tissues by loading the composite material is almost complete after 8 weeks.

In this study, we clearly showed that CB‐bFGF/CP promoted bone regeneration and suppressed the downgrowth of epithelial tissues in the rat horizontal bone defect model. This result indicates the possibility that application of bFGF can be expanded to horizontal bone loss by anchoring to collagen scaffold. CB‐bFGF composite material may play a role in the provision of space and stimulation of mesenchymal stem cells derived from periodontal ligament. In addition, the improvement in local retention of CB‐bFGF due to the binding to collagen scaffolds may contribute to enhance the efficacy. There is also a possibility that CB‐bFGF binds to collagen fibers such as alveolar bone, dentin, cementum, and ECM, after being released from collagen scaffolds to enhance the efficacy. In addition to bone regeneration, formations of a new cementum and a new periodontal ligament are required for periodontal regeneration. Further investigation, e.g., evaluation in larger animals, is necessary to evaluate the efficacy of CB‐bFGF composite material for periodontal regenerative. It is also necessary to reveal the dynamics of CB‐bFGF in the local tissue in vivo. CB‐bFGF composite material is expected to be an improved therapeutic material as a novel drug delivery system for periodontal regeneration.

## 5 CONCLUSIONS

The efficacy of CB‐bFGF with collagen scaffolds was superior to bFGF in bone regeneration and suppression of epithelial downgrowth at horizontal bone defect in rats. The results suggest that the various functions of bFGF are enhanced by improving the local retention using collagen‐anchoring. This finding suggests that CB‐bFGF composite material is a potential candidate for periodontal regeneration as a novel drug delivery system.

## Supporting information

FigureS1

FigureS2

**FIGURE S1**.Binding of the purified CB‐bFGF to CP. Collagen binding assay was performed in the presence and absence of CP. M; Molecular weight markers, Lane 1; CP, Lane 2; CB‐bFGF, Lane 3; CB‐bFGF + CP, Lane 4; bFGF, Lane 5; bFGF + CP
**FIGURE S2**.The confirmation of fluorescent label of proteins by Alexa Fluor 594 dye (Lane 1&2; CB‐bFGF, Lane 3&4; bFGF). Collagen binding assay of the labeled proteins (Lane 5–7; CB‐bFGF, Lane 8–10; bFGF). Unreacted dye was located at the lowest position of the gels.

## Footnotes

1 CLEA, Tokyo, Japan.

2
*Escherichia coli* BL21 CodonPlus RIL, Agilent Technologies, Santa Clara, CA.

3 Glutathione Sepharose 4B, GE Healthcare Japan, Tokyo, Japan.

4 GE Healthcare Japan, Tokyo, Japan.

5 GE Healthcare Japan, Tokyo, Japan.

6 Funakoshi, Tokyo, Japan.

7 Sanko Junyaku, Chiba, Japan.

8 Thermo Fisher Scientific, Waltham, MA.

9 Sigma‐Aldrich, St. Louis, MO.

10 Cell Counting Kit‐8, Dojindo Laboratories, Kumamoto, Japan.

11 Wako, Tokyo, Japan.

12 Alexa Fluor 594 dye, Thermo Fisher Scientific, Waltham, MA.

13 SpinTrap column, GE Healthcare Japan, Tokyo, Japan.

14 ChemiDoc MP System, BioRad Laboratories, Hercules, CA.

15 IVIS Lumina, Sumisho Pharmaceutical International, Tokyo, Japan.

16 ImageJ, National Institutes of Health, Bethesda, MD.

17 Beldenta Supply, Hyogo, Japan.

18 Nacalai Tesque, Kyoto, Japan.

19 inspeXio SMX‐90CT, Shimazu, Kyoto, Japan.

20 Tri‐3D‐Bone, Ratoc System Engineering, Tokyo, Japan.

21 ImageJ, National Institutes of Health, Bethesda, MD.

22 VECTASTAIN *Elite* ABC Rabbit kit, Vector Laboratories, Burlingame, CA.

23 Thermo Fisher Scientific, Waltham, MA.

24 BIOSS, Woburn, MA.

25 Gene Tex, Irvine, CA.

26 JMP (Ver. 9.0.2), SAS Institute, Cary, NC.

27 Nacalai Tesque, Kyoto, Japan.
