# Supplementary material for: Acceleration of bone regeneration of horizontal bone defect in rats using collagen‐binding basic fibroblast growth factor combined with collagen scaffolds
Source: J Periodontol. 2019 Apr 14;90(9):1043–52. doi: 10.1002/JPER.18-0674 (PMC6850180; doi:10.1002/JPER.18-0674)
Supplement: Supplementary file 1 — FigureS1 [file JPER-90-1043-s001.pdf]

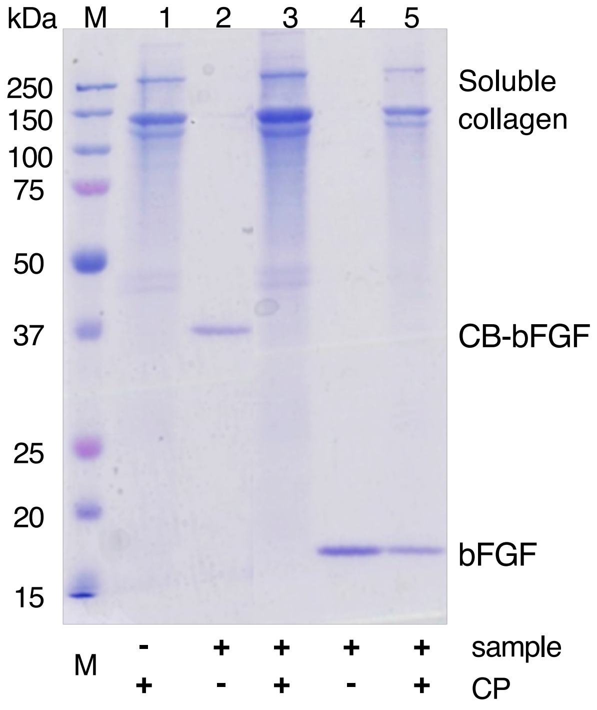

FIGURE S1. Binding of the purified CB-bFGF to collagen powder (CP). Collagen binding assay was performed in the presence and absence of CP. M; Molecular weight markers, Lane 1; CP, Lane 2; CB-bFGF, Lane 3; CB-bFGF + CP, Lane 4; bFGF, Lane 5; bFGF + CP
