# Supplementary material for: Acceleration of bone regeneration of horizontal bone defect in rats using collagen‐binding basic fibroblast growth factor combined with collagen scaffolds
Source: J Periodontol. 2019 Apr 14;90(9):1043–52. doi: 10.1002/JPER.18-0674 (PMC6850180; doi:10.1002/JPER.18-0674)
Supplement: Supplementary file 2 — FigureS2 [file JPER-90-1043-s002.pdf]

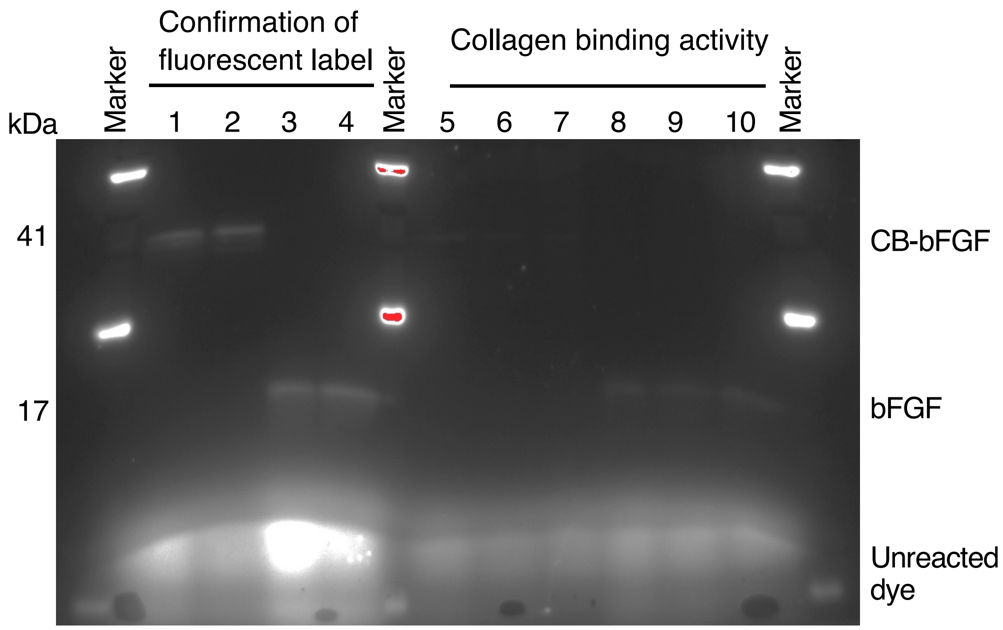

FIGURE S2. The confirmation of fluorescent label of proteins by Alexa Fluor 594 dye (Lane 1&2; CB-bFGF, Lane 3&4; bFGF). Collagen binding assay of the labeled proteins (Lane 5–7; CB-bFGF, Lane 8–10; bFGF). Unreacted dye was located at the lowest position of the gels.
