# Supplementary material for: Acceleration of bone regeneration of horizontal bone defect in rats using collagen‐binding basic fibroblast growth factor combined with collagen scaffolds
Source: J Periodontol. 2019 Apr 14;90(9):1043–52. doi: 10.1002/JPER.18-0674 (PMC6850180; doi:10.1002/JPER.18-0674)
Supplement: Supplementary file 3 — FIGURE S1.Binding of the purified CB‐bFGF to CP. Collagen binding assay was performed in the presence and absence of CP. M; Molecular weight markers, Lane 1; CP, Lane 2; CB‐bFGF, Lane 3; CB‐bFGF + CP, Lane 4; bFGF, Lane 5; bFGF + CP FIGURE S2.The confirmation of fluorescent label of proteins by Alexa Fluor 594 dye (Lane 1&2; CB‐bFGF, Lane 3&4; bFGF). Collagen binding assay of the labeled proteins (Lane 5–7; CB‐bFGF, Lane 8–10; bFGF). Unreacted dye was located at the lowest position of the gels. [file JPER-90-1043-s003.docx]

**Supporting information**

***FIGURE S1.***

Binding of the purified CB-bFGF to collagen powder (CP). Collagen binding assay was performed in the presence and absence of CP. M; Molecular weight markers, Lane 1; CP, Lane 2; CB-bFGF, Lane 3; CB-bFGF + CP, Lane 4; bFGF, Lane 5; bFGF + CP

***FIGURE S2.***

The confirmation of fluorescent label of proteins by Alexa Fluor 594 dye (Lane 1&2; CB-bFGF, Lane 3&4; bFGF). Collagen binding assay of the labeled proteins (Lane 5–7; CB-bFGF, Lane 8–10; bFGF). Unreacted dye was located at the lowest position of the gels.
